# Stress-Related Disorders Among Young Individuals With Surgical Removal of Tonsils or Adenoids

**DOI:** 10.1001/jamanetworkopen.2024.49807

**Published:** 2024-12-09

**Authors:** Xue Xiao, Fen Yang, Li Yin, Josef Isung, Weimin Ye, David Mataix-Cols, Zhe Zhang, Unnur Valdimarsdóttir, Fang Fang

**Affiliations:** 1 Department of Otolaryngology–Head and Neck Surgery, First Affiliated Hospital of Guangxi Medical University, Nanning, Guangxi, China; 2 Key Laboratory of Early Prevention and Treatment for Regional High-Frequency Tumor (Guangxi Medical University), Ministry of Education and Guangxi, Nanning, Guangxi, China; 3 Institute of Environmental Medicine, Karolinska Institutet, Stockholm, Sweden; 4 Department of Medical Epidemiology and Biostatistics, Karolinska Institutet, Stockholm, Sweden; 5 Centre for Psychiatry Research, Department of Clinical Neuroscience, Karolinska Institutet and Stockholm Health Care Services, Stockholm County Council, Stockholm, Sweden; 6 Department of Clinical Sciences, Lund University, Lund, Sweden; 7 Center of Public Health Sciences, Faculty of Medicine, University of Iceland, Reykjavík, Iceland; 8 Department of Epidemiology, Harvard T. H. Chan School of Public Health, Boston, Massachusetts

## Abstract

**Importance:**

Studies have suggested an increased risk of psychiatric disorders and suicidal behavior among individuals who have undergone tonsillectomy. However, little is known about stress-related disorders.

**Objective:**

To investigate whether surgical removal of tonsils or adenoids is associated with a subsequent risk of stress-related disorders.

**Design, Setting, and Participants:**

This cohort study used Swedish nationwide population and health registry data of all individuals born between January 1, 1981, and December 31, 2016. Individuals who had undergone surgical removal of tonsils or adenoids (ie, exposed persons) were compared with unrelated unexposed individuals individually matched by sex, birth year, and calendar date at the start of follow-up (population-matched cohort) and with their unexposed full siblings (sibling-matched cohort). The analysis was performed between December 15, 2023, and October 11, 2024.

**Exposure:**

Surgical removal of tonsils or adenoids.

**Main Outcomes and Measures:**

The Swedish Patient Register was used to identify stress-related disorders, including posttraumatic stress disorder (PTSD), acute stress reaction, and adjustment disorder or other stress reaction. In the population-matched cohort, a Cox proportional hazards regression model was used to assess the association of tonsillectomy with the risk of stress-related disorders, conditioned on sex, birth year, and calendar date at the start of follow-up and adjusted for parental educational attainment and history of stress-related disorders. To address potential familial confounding, analyses were replicated using the sibling-matched cohort.

**Results:**

The population-matched cohort included 83 957 exposed and 839 570 unexposed persons (median [IQR] age at the start of follow-up, 14.4 [6.5-18.6] years; 55.2% female), and the sibling-matched cohort included 51 601 exposed persons (median [IQR] age at start of follow-up, 14.9 [6.9-18.7] years, 55.8% female) and 75 159 unexposed full siblings (median [IQR] age at start of follow-up, 13.3 [6.9-19.5] years; 52.6% male). Compared with the unexposed population reference, exposed persons exhibited a higher subsequent risk of stress-related disorders (hazard ratio [HR], 1.43; 95% CI, 1.38-1.48), especially PTSD (HR, 1.55; 95% CI, 1.43-1.69). These results were replicated in the sibling-matched cohort (any stress-related disorder: HR, 1.34 [95% CI, 1.25-1.44]; PTSD: HR, 1.41 [95% CI, 1.18-1.69]). An increased risk was consistently noted regardless of sex, age at surgery, time since surgery, parental educational attainment, or parental history of stress-related disorders and was mainly noted for a surgery due to adenotonsillar diseases or sleep and respiratory abnormalities.

**Conclusions and Relevance:**

In this cohort study, the findings suggest that early-life surgical removal of tonsils or adenoids is associated with a higher future risk of stress-related disorders and highlight a need to understand the role of adenotonsillar diseases or associated health conditions in the development of stress-related psychiatric disorders.

## Key Points

**Question:**

Is undergoing surgical removal of tonsils or adenoids in early life associated with increased subsequent risk of stress-related disorders?

**Findings:**

In this cohort study of 1 050 287 children and young adults in Sweden, those who underwent surgical removal of tonsils or adenoids exhibited a higher risk of stress-related disorders, especially posttraumatic stress disorder, compared with unexposed individuals or unexposed full siblings.

**Meaning:**

These findings suggest a potential role of adenotonsillar diseases or associated health conditions in the development of stress-related disorders.

## Teaser

This cohort study investigates whether surgical removal of tonsils or adenoids is associated with a subsequent risk of stress-related disorders among children and young adults.

## Introduction

Tonsillectomy is a widely performed surgical procedure, often for recurrent tonsillitis, peritonsillar abscess, and obstructive sleep-disordered breathing among children and for obstructive sleep apnea and suspicion for a malignant neoplasm among adults.^1^ In the US, nearly 300 000 children undergo this procedure annually.^2^ In Sweden, approximately 13 500 individuals undergo tonsillectomy annually.^3^ Given its high prevalence, especially in early life, it is important to understand the long-term health outcomes of children undergoing tonsillectomy. Individuals who have undergone tonsillectomy have been found to have an increased risk of irritable bowel syndrome^4^; autoimmune,^5^ respiratory, allergic, and infectious diseases^6^; premature myocardial infarction^7^; and some cancer types.^8^ Several reasons might underlie such findings. First, indications of tonsillectomy, eg, repeated or persistent pharyngeal infections and resultant inflammation, might lead to an altered risk of later health outcomes. Second, the removal of tonsils leads to physiologic changes that might be of relevance to future disease risk. For example, removal of the tonsils eliminates the first-line defense against ingested and inhaled pathogens and may lead to altered immune responses and subsequent disease risk.^9^ Finally, there may be common causes for both a need for tonsillectomy in early life and an increased risk of adverse health outcomes later in life, ie, confounding bias.

Psychiatric disorders are among the most common causes of morbidity today^10^ and typically emerge in childhood, adolescence, or early adulthood.^11^ A previous study found an increased risk of multiple psychiatric disorders and suicidal behavior among individuals with tonsillectomy and hypothesized that chronic inflammation within the mucosa-associated lymphoid tissue may be an underlying mechanism.^12^ Although relatively little is known regarding the intersection between health conditions of the head and neck and psychiatric disorders, or the potential role of chronic inflammation in such an intersection, studies have shown that individuals with chronic inflammatory conditions of the head and neck, including rhinosinusitis and otitis media, may have an increased risk of depression, anxiety, and stress-related disorders.^13,14^ A few smaller studies have also suggested an increased risk of psychiatric disorders or symptoms among children with tonsillectomy, hypothesizing that indications of tonsillectomy and presurgery temperament might be some of the potential explanations.^15,16,17,18^

In contrast to other psychiatric disorders, little is known about the risk of stress-related disorders, including posttraumatic stress disorder (PTSD), acute stress reaction, adjustment disorder, and other stress reaction, following tonsillectomy. As stress-related disorders are common in our society, it is important to understand their causes and risk factors for better prevention and treatment strategies. To this end, we conducted a nationwide matched cohort study in Sweden to assess the risk of stress-related disorders among children (aged <18 years) and young adults (aged 19-36 years) who have undergone previous surgical removal of the tonsils or adenoids compared with individuals who have not.

## Methods

### Study Design

In this cohort study, we first created a study base using data from the Swedish Total Population Register, including all individuals born between January 1, 1981, through December 31, 2016, in Sweden whose parents were also born in Sweden. We followed these individuals from birth until surgical removal of tonsils or adenoids, emigration, death, or December 31, 2016, whichever came first. The individual follow-up was made possible through cross linkage to the Swedish Patient Register, Total Population Register, and Causes of Death Register using the individually unique personal identification numbers. After excluding individuals with conflicting information, the study base included 3 811 283 individuals. The statistical analysis was conducted between December 15, 2023, and October 11, 2024. This study was approved by the Swedish Ethical Review Authority. A requirement for informed consent was waived by this approval given the register-based nature of the study. This study followed the Strengthening the Reporting of Observational Studies in Epidemiology (STROBE) reporting guideline.

Within the study base, we formed a population-matched cohort using the exposure density sampling technique.^19^ For a person who underwent surgical removal of tonsils or adenoids (ie, exposed person), we randomly selected 10 unexposed persons who were free of the exposure on the date of surgery and individually matched them on sex and birth year. We also conducted a sibling-matched cohort to address potential familial confounding. Full siblings share, on average, 50% of their genetic factors and many environmental factors during their upbringing. As a result, a comparison of differentially exposed full siblings implicitly adjusts for potential confounding due to such shared factors.^20^ We linked all individuals included in the study base to the Swedish Multi-Generation Register, which includes information on familial links for people born since 1932,^21^ to identify full siblings. For each exposed person, we included all full siblings who were alive and free of the exposure on the date of surgery as the unexposed siblings.

In both the population- and sibling-matched cohorts, we followed the exposed person and their individually matched unexposed persons (or siblings) from date of surgery (ie, start of follow-up) until the first diagnosis of a stress-related disorder, emigration, death, or end of study, whichever came first. For the unexposed persons (or siblings), follow-up was also censored in case they experienced the surgery during follow-up, after which time, they became an exposed person and were matched individually to unexposed persons (or siblings). Persons with a previous diagnosis of stress-related disorders before the start of follow-up were excluded from the analysis.

### Exposure

Although tonsillectomy and adenoidectomy are separate surgeries with different indications, they are often performed concurrently. We therefore identified a surgical removal of tonsils or adenoids, namely tonsillectomy, adenotonsillectomy, or adenoidectomy, from the Patient Register, which has collected nationwide information on hospital-based inpatient care since 1987 and outpatient care since 2001, including discharge diagnoses and surgical procedures.^22^ Surgical removal of tonsils or adenoids was exclusively performed via inpatient care until 2006 in Sweden^23^; we therefore identified such procedures from both inpatient and outpatient care. We used surgical codes 2710 before 1997 and EMB10 since 1997 to identify tonsillectomy, 2720 before 1997 and EMB20 since 1997 to identify adenotonsillectomy, and 2730 before 1997 and EMB30 since 1997 to identify adenoidectomy.

### Outcome

A first diagnosis of stress-related disorder during follow-up was identified from the Patient Register. Although coverage for all inpatient care was nationwide starting in 1987, the register covers inpatient care for psychiatric disorders since 1973. During the follow-up, we obtained complete information on inpatient-based diagnoses of stress-related disorders until 2000 and on both inpatient- and outpatient-based diagnoses from 2001 onward. We identified any stress-related disorder using *International Classification of Diseases, Revision 8* (*ICD-8*) codes 307 and 308.4; *International Classification of Diseases, Ninth Revision* (*ICD-9*) codes 308 and 309; and *International Statistical Classification of Diseases, Tenth Revision* (*ICD-10*) code F43. We also studied PTSD (*ICD-9* code 309B, *ICD-10* code F43.1), acute stress reaction (*ICD-9* codes 308 and 309A, *ICD-10* code F43.0), and adjustment disorder or other stress reaction (*ICD-9* code 309X, *ICD-10* codes F43.2, F43.8, and F43.9) separately. As the subtypes were only possible to differentiate in *ICD-9* and *ICD-10*, this analysis was conducted from 1987 onward. Diagnostic codes within the Patient Register have a positive predicted value of 85% to 95% for most common diseases^22^ and 75% to 90% for PTSD.^24^

### Covariates

We used age, sex, calendar period, parental educational attainment (as a proxy for early-life socioeconomic status), and parental history of stress-related disorders (as a proxy for genetic predisposition to stress-related disorders) as covariates. We identified the parents of the study participants through the Multi-Generation Register and linked them to the Swedish Longitudinal Integrated Database for Health Insurance and Labor (LISA) to obtain information on the highest parental educational attainment. Since 1990, LISA has collected annually updated data on demographic and socioeconomic status for individuals aged 16 years or older in Sweden. We also linked the parents to the Patient Register to identify history of stress-related disorders.

### Statistical Analysis

We first summarized characteristics of the study participants by exposure status. We then used Kaplan-Meier survival curves to compare the cumulative incidence of stress-related disorders between the exposed and unexposed groups using the servicer package in R, version 3.6.0 (R Foundation). We also calculated the crude incidence rate (IR) of stress-related disorders, dividing the number of incident cases by accumulated person-years.

In the population-matched cohort, we used a conditional Cox proportional hazards regression model to estimate the mean hazard ratio (HR) and 95% CI of stress-related disorders in relation to the exposure. We used time since the start of follow-up as the underlying timescale and adjusted for parental educational attainment and parental history of stress-related disorders. Given the use of individual match and exposure density sampling, sex, birth year, and calendar date at the start of follow-up were inherently adjusted for in the analysis. We performed several sensitivity analyses to alleviate potential concern regarding surveillance bias (ie, individuals who underwent a surgical procedure might be under more surveillance than others, leading to a higher-than-expected detection for stress-related disorders) by excluding the first 1, 2, or 3 years of follow-up from the analysis. We evaluated the proportional hazards assumption using Schoenfeld residual test (R function cox.zph), which indicates a violated assumption in both cohorts. For the sake of simplicity, we present 1 overall HR for the entire follow-up for most of the analyses; however, we also conducted a stratified analysis by time at follow-up to show the varying HRs.

We analyzed any stress-related disorder as well as PTSD, acute stress reaction, and adjustment disorder or other stress reaction. We then focused on any stress-related disorder as the outcome and conducted stratified analyses by sex, age at start of follow-up, time since start of follow-up, parental educational attainment, and parental history of stress-related disorders. We included an interaction term with the exposure and a specific covariate in the model to evaluate whether the association was modified by the covariate. For the exposed persons, we identified from the Patient Register any hospital visit concerning hypertrophy of the tonsils and adenoids, chronic infection in the tonsils or adenoids, other chronic diseases of the tonsils or adenoids, or sleep-related conditions before the surgery and considered them as potential indications for surgery (eTable 1 in Supplement 1). We then conducted separate analyses for surgeries with different potential indications.

We replicated these analyses in the sibling-matched cohort using Cox proportional hazards regression models conditioned on family identifiers, with time since start of follow-up as the underlying timescale and adjustment for sex and age at the start of follow-up. Parental educational attainment and history of stress-related disorders were not adjusted for as they were shared between full siblings. To test the hypothesis that removal of tonsils or adenoids may lead to an increased risk of stress-related disorders through an altered risk of infections, we conducted a sensitivity analysis to assess risk of hospital-treated infectious diseases, ie, first hospital visit for which an infectious disease was indicated as the primary diagnosis during follow-up based on *ICD* codes.^25^ Finally, to test the hypothesis that temperament or susceptibility to psychiatric disorders in general might be an explanation for the association, we conducted another sensitivity analysis to assess risk of other psychiatric disorders (*ICD-8* codes 290-315, *ICD-9* codes 290-319, and *ICD-10* codes F00-F99, excluding codes for stress-related disorders) following the surgery.

Data management and analyses were conducted using SAS, version 9.4 (SAS Institute Inc) and R, version 3.6.0. A 2-sided *P* < .05 was considered statistically significant. We did not adjust for multiplicity of statistical tests, as adopting a top-down approach, the main hypothesis of an increased risk of stress-related disorders after surgical removal of tonsils or adenoids, consists of only 1 test.

## Results

The population-matched cohort included 83 957 exposed and 839 570 unexposed persons (median [IQR] age, 14.4 [6.5-18.6] years; 55.2% female and 44.8% male), whereas the sibling-matched cohort included 51 601 exposed persons (median [IQR] age at start of follow-up, 14.9 [6.9-18.7] years, 55.8% female and 44.2% male) and 75 159 unexposed full siblings (median [IQR] age at start of follow-up, 13.3 [6.9-19.5] years; 47.4% female and 52.6% male). In the population-matched cohort, exposed persons had lower parental educational attainment and a higher prevalence of parental history of stress-related disorders (Table 1). These differences disappeared or diminished in the sibling-matched cohort. In both cohorts, exposed persons had a higher cumulative incidence of stress-related disorders compared with unexposed persons (or siblings) throughout follow-up (Figure).

**Table 1.** Baseline Characteristics of the Study Participants by Status of Surgical Removal of Tonsils or Adenoids

Characteristic	No. (%)			
	Population-matched cohort		Sibling-matched cohort	
	Individuals with surgery (n = 83 957)	Unexposed individuals (n = 839 570)^a^	Individuals with surgery (n = 51 601)	Unexposed siblings (n = 75 159)^a^
Age at start of follow-up, median (IQR), y	14.4 (6.5-18.6)	14.4 (6.5-18.6)	14.9 (6.9-18.7)	13.3 (6.9-19.5)
Sex				
Female	46 311 (55.2)	463 110 (55.2)	28 772 (55.8)	35 616 (47.4)
Male	37 646 (44.8)	376 460 (44.8)	22 829 (44.2)	39 543 (52.6)
Highest level of parental education, y				
≤9	5069 (6.0)	45 840 (5.5)	2439 (4.7)	3900 (5.2)
>9-12	41 250 (49.1)	351 065 (41.8)	26 946 (52.2)	39 026 (51.9)
>12	30 432 (36.3)	326 634 (38.9)	22 206 (43.0)	31 192 (41.5)
Unknown	7206 (8.6)	116 031 (13.8)	10 (0.0)	1041 (1.4)
Parental history of stress-related disorders				
Yes	3598 (4.3)	28 826 (3.4)	2240 (4.3)	3506 (4.7)
No	80 359 (95.7)	810 744 (96.6)	49 361 (95.7)	71 653 (95.3)

^a^ No surgical removal of tonsils or adenoids.

**Figure.**
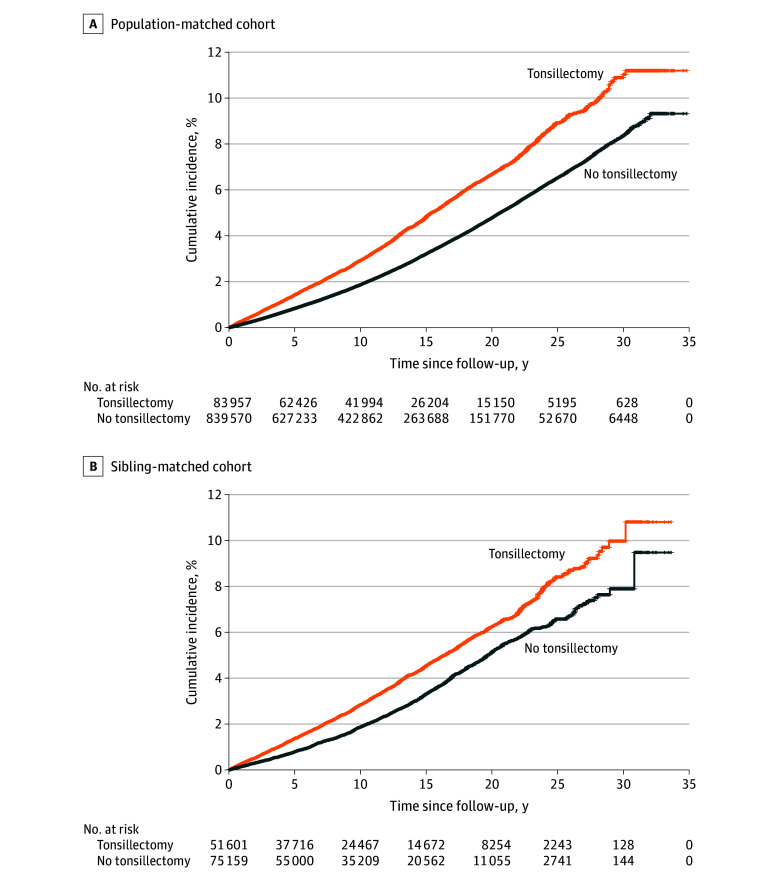
Cumulative Incidence of Stress-Related Disorders

In the population-matched cohort, we observed an IR of 33.3 per 10 000 person-years for any stress-related disorders among exposed persons compared with an IR of 22.7 per 10 000 person-years among unexposed persons, leading to an HR of 1.43 (95% CI, 1.38-1.48) during follow-up (Table 2). Similar results were noted for PTSD, acute stress reaction, and adjustment disorder or other stress reaction, although the risk for PTSD was greater (HR, 1.55; 95% CI, 1.43-1.69). In the sibling-matched cohort, we observed similar associations (any stress-related disorder: HR, 1.34 [95% CI, 1.25-1.44]; PTSD: HR, 1.41 [95% CI, 1.18-1.69]). Incorporating a lag period of different lengths did not lead to different results (eTable 2 in Supplement 1).

**Table 2.** Incidence and Risk of Stress-Related Disorders Associated With Surgical Removal of Tonsils or Adenoids

Matched cohort	Individuals without surgery			Individuals with surgery		
	No. of cases	IR per 10 000 person-y	HR (95% CI)^a^	No. of cases	IR per 10 000 person-y	HR (95% CI)^a^
Population						
Any stress-related disorder	21 862	22.7	1 [Reference]	3194	33.3	1.43 (1.38-1.48)
Posttraumatic stress disorder	4007	4.1	1 [Reference]	638	6.6	1.55 (1.43-1.69)
Acute stress reaction	10 581	11.0	1 [Reference]	1600	16.7	1.47 (1.39-1.55)
Adjustment disorder or other stress reaction	10 274	10.7	1 [Reference]	1477	15.4	1.41 (1.34-1.49)
Sibling						
Any stress-related disorder	1810	22.5	1 [Reference]	1742	31.0	1.34 (1.25-1.44)
Posttraumatic stress disorder	334	4.2	1 [Reference]	362	6.4	1.41 (1.18-1.69)
Acute stress reaction	894	11.1	1 [Reference]	860	15.3	1.36 (1.22-1.51)
Adjustment disorder or other stress reaction	859	10.7	1 [Reference]	797	14.2	1.25 (1.12-1.39)

Abbreviations: HR, hazard ratio; IR, incidence rate.

^a^ Analysis of the population-matched cohort used time since follow-up as the underlying timescale and was conditioned on sex, birth year, and calendar date at the start of follow-up and additionally adjusted for parental educational attainment and parental history of stress-related disorders at the start of follow-up. Analysis of the sibling-matched cohort used time since follow-up as the underlying timescale and was conditioned on family identifiers and calendar date at the start of follow-up and additionally adjusted for sex and age at the start of follow-up. Parental educational attainment and history of stress-related disorders at the start of follow-up were not adjusted for in the sibling-matched cohort as the exposed person and their unexposed full siblings shared this information at the start of follow-up.

Stratified analyses of the population-matched cohort showed a consistently increased risk of stress-related disorders associated with surgical removal of tonsils or adenoids, regardless of sex, age at surgery, time since surgery, parental educational attainment, or parental history of stress-related disorders (Table 3). Risks were observed to increase with increasing age at surgery (26 years or older: HR, 1.76; 95% CI, 1.43-2.15; *P* for interaction <.001) and to decrease with increasing time since surgery (0-10 years after surgery: HR 1.55; 95% CI, 1.47-1.63; *P* for interaction <.001). The risk was also slightly higher among women than men (*P* for interaction = .01) but did not vary by parental educational attainment (*P* for interaction = .07) or history of stress-related disorders (*P* for interaction = .96). The sibling-matched cohort showed similar results (eTable 3 in Supplement 1). An increased risk of stress-related disorders was noted for different indications for surgery in both cohorts, notably adenotonsillar diseases or sleep and respiratory abnormalities (eTable 4 in Supplement 1).

**Table 3.** Incidence and Risk of Stress-Related Disorders Associated With Surgical Removal of Tonsils or Adenoids in the Population-Matched Cohort, Stratified Analysis

Characteristic	Individuals without surgery			Individuals with surgery			
	No. of cases	IR per 10 000 person-y	HR (95% CI)^a^	No. of cases	IR per 10 000 person-y	HR (95% CI)^a^	*P* value for interaction
Sex							
Male	6379	13.6	1 [Reference]	859	18.4	1.31 (1.22-1.41)	.01
Female	15 483	31.2	1 [Reference]	2335	47.4	1.47 (1.41-1.54)	
Age at the start of follow-up, y							
1-6	4005	13.0	1 [Reference]	540	17.5	1.32 (1.20-1.44)	<.001
7-12	5005	18.1	1 [Reference]	670	24.2	1.31 (1.21-1.42)	
13-18	7085	31.7	1 [Reference]	1150	52.2	1.60 (1.50-1.70)	
19-25	5142	36.7	1 [Reference]	724	52.2	1.41 (1.31-1.53)	
≥26	625	40.0	1 [Reference]	110	71.2	1.76 (1.43-2.15)	
Time since the start of follow-up, y							
0-10	11 524	11.9	1 [Reference]	1841	19.1	1.55 (1.47-1.63)	<.001
11-20	7985	10.5	1 [Reference]	1068	14.2	1.32 (1.24-1.41)	
≥21	2351	6.4	1 [Reference]	284	7.7	1.19 (1.05-1.35)	
Parental educational attainment, y							
≤9	1652	35.2	1 [Reference]	261	50.1	1.38 (1.24-1.55)	.07
>9-12	10 838	25.8	1 [Reference]	1740	37.3	1.43 (1.36-1.50)	
>12	7729	18.1	1 [Reference]	1004	27.1	1.51 (1.40-1.62)	
Parental history of stress-related disorders							
No	18 245	20.4	1 [Reference]	2548	29.2	1.43 (1.37-1.48)	.96
Yes	3617	51.3	1 [Reference]	646	73.5	1.42 (1.23-1.62)	

Abbreviations: HR, hazard ratio; IR, incidence rate.

^a^ Analysis of the population-matched cohort used time since follow-up as the underlying timescale and was conditioned on sex, birth year, and calendar date at the start of follow-up and additionally adjusted for parental educational attainment (except for the stratified analysis by parental educational attainment) and parental history of stress-related disorders (except for the stratified analysis by parental history of stress-related disorders).

In the sensitivity analyses, we observed an HR of 1.61 (95% CI, 1.59-1.63) in the population-based cohort and an HR of 1.31 (95% CI, 1.29-1.33) in the sibling-matched cohort for hospital-treated infectious diseases (eTable 5 in Supplement 1). The corresponding HRs were 1.31 (95% CI, 1.28-1.34) and 1.24 (95% CI, 1.20-1.27) for other psychiatric disorders.

## Discussion

In this nationwide matched cohort study of children and young adults in Sweden, we found that having undergone surgical removal of the tonsils or adenoids was associated with a higher future risk of stress-related disorders. An increased risk was consistently noted, regardless of sex, age at surgery, time since surgery, parental educational attainment (proxy for early-life socioeconomic status), parental history of stress-related disorders (proxy of predisposition to stress-related disorders), or potential indication for the surgery.

Although the literature is expanding on health outcomes of individuals undergoing tonsillectomy, relatively little is known regarding psychiatric disorders. Several earlier studies, primarily case reports and case series, have shown an increased risk of psychiatric disorders or prevalence of psychiatric symptoms among individuals who had undergone tonsillectomy,^15,16,17^ although another study reported no change in psychological status at 3 weeks after tonsillectomy.^26^ In a previous study, we showed an increased risk of multiple psychiatric disorders and suicidal behavior among individuals with tonsillectomy compared with an unrelated population or siblings.^12^

Several explanations have been proposed for an increased risk of psychiatric disorders following tonsillectomy. The reason for the surgery, such as chronic throat infections, may lead to persistent inflammation within the mucosa-associated lymphoid tissue of the tonsils and, subsequently, to an alteration in immune responses and changes in defense against constant exposure to pathogens and inflammatory challenges.^12^ Similarly, the surgery itself eliminates the first-line defense against ingested and inhaled pathogens, as partly evidenced by the increased risk of hospital-treated infectious diseases noted among the exposed persons in our study. Evidence has indeed accumulated to support chronic inflammation as a pivotal molecular basis in the pathogenesis of psychiatric disorders,^27^ including stress-related disorders.^20^ For instance, peripheral inflammatory mediators, including cytokines, may reach the central nervous system and induce neuroinflammation, subsequently impairing neuronal plasticity and neurochemistry and modulating neuroendocrine axes.^27^

The short-term risk increase in psychiatric disorders or symptoms may be a result of hospitalization, separation from attachment figures, anesthetic procedure, surgery, posttonsillectomy experience, or other reasons that could resolve over time.^15^ However, we found that although the risk increase appeared to be greatest during the first years following surgery, an increased risk of stress-related disorders was still noted more than 20 years after the surgery. The greater risk increase noted among individuals who had undergone surgery at an older age may be partially attributed to the recency of their exposure. Furthermore, individuals who need such surgery may be at a higher risk of psychiatric disorders due to preexisting emotional and behavioral problems.^18^ We found that the exposed persons had a higher risk of any psychiatric disorder (apart from stress-related disorders) following the surgery, which is in line with our group’s previous report.^12^ Regardless, if our findings here are validated in future studies of independent study populations, mechanistic studies would be needed to disentangle the role of human tonsils and their diseases, via inflammation or other associated health conditions, in the development of psychiatric disorders in general and stress-related disorders specifically.

### Strengths and Limitations

Our study has several strengths, including the nationwide design; large sample size; complete follow-up; and prospective and independent ascertainment of exposure, outcome, and covariates, alleviating greatly the concern of most systematic and random errors. Being able to contrast findings between a population-matched cohort and a sibling-matched cohort is another strength and helped to allay concern regarding bias due to familial confounding.

Our study also has several limitations. First, given the registry-based nature of the study, we did not have access to medical records, preventing the possibility of studying clinical characteristics of the surgery. It remains interesting to examine whether the increased risk of stress-related disorders varies by timeliness of surgery or by whether there is a complete remission of symptoms after surgery. Second, as we identified stress-related disorders through specialist care, our findings are not directly generalizable to stress-related disorders attended by primary care. Third, although the diagnosis of PTSD has satisfactory quality, validation studies are lacking for other stress-related disorders in the Patient Register. Fourth, although we conducted multivariable adjustment, stratified analysis, and sibling comparison to eliminate confounding, we could not rule out the possibility of residual confounding due to factors not shared between siblings (eg, pubertal changes, adult lifestyle factors). Finally, although we assumed that full siblings shared lifestyle and environmental factors during childhood, we had little knowledge about whether they lived together during childhood.

## Conclusions

In this cohort study of children and young adults in Sweden, individuals who underwent surgical removal of tonsils or adenoids had a higher future risk of stress-related disorders compared with an unrelated population reference or with their unexposed full siblings. This association was independent of age at the time of surgery or time since surgery, sociodemographic or medical characteristics of the patients, and familial confounding.

## Notes

Supplement 1. **eTable 1.** Swedish Revisions of the *International Classification of Diseases* (*ICD*) Codes Used to Identify the Indications for Surgical Removal of Tonsils or Adenoids **eTable 2.** Incidence Rate (IR, per 10 000 Person-Years) and Hazard Ratio (HRs) With 95% CIs of Stress-Related Disorders Associated With Surgical Removal of Tonsils or Adenoids, Sensitivity Analyses With Different Lag Periods **eTable 3.** Incidence Rate (IR, per 10 000 Person-Years) and Hazard Ratio (HRs) With 95% CIs of Stress-Related Disorders Associated With Surgical Removal of Tonsils or Adenoids in the Sibling-Matched Cohort, Stratified Analysis **eTable 4.** Incidence Rate (IR, per 10 000 Person-Years) and Hazard Ratio (HRs) With 95% CIs of Stress-Related Disorders Associated With Surgical Removal of Tonsils or Adenoids in the Population-Matched and Sibling-Matched Cohorts, Analysis by Potential Indication for Surgery **eTable 5.** Incidence Rate (IR, per 10 000 Person-Years) and Hazard Ratio (HRs) With 95% CIs of Hospital-Treated Infectious Diseases or Other Psychiatric Disorders Associated With Surgical Removal of Tonsils or Adenoids in the Population-Matched and Sibling-Matched Cohorts

Supplement 2.
Data Sharing Statement
