# Supplementary material for: Stress-Related Disorders Among Young Individuals With Surgical Removal of Tonsils or Adenoids
Source: JAMA Netw Open. 2024 Dec 9;7(12):e2449807. doi: 10.1001/jamanetworkopen.2024.49807 (PMC11629130; doi:10.1001/jamanetworkopen.2024.49807)
Supplement: Supplement 1. — eTable 1. Swedish Revisions of the International Classification of Diseases (ICD) Codes Used to Identify the Indications for Surgical Removal of Tonsils or Adenoids eTable 2. Incidence Rate (IR, per 10 000 Person-Years) and Hazard Ratio (HRs) With 95% CIs of Stress-Related Disorders Associated With Surgical Removal of Tonsils or Adenoids, Sensitivity Analyses With Different Lag Periods eTable 3. Incidence Rate (IR, per 10 000 Person-Years) and Hazard Ratio (HRs) With 95% CIs of Stress-Related Disorders Associated With Surgical Removal of Tonsils or Adenoids in the Sibling-Matched Cohort, Stratified Analysis eTable 4. Incidence Rate (IR, per 10 000 Person-Years) and Hazard Ratio (HRs) With 95% CIs of Stress-Related Disorders Associated With Surgical Removal of Tonsils or Adenoids in the Population-Matched and Sibling-Matched Cohorts, Analysis by Potential Indication for Surgery eTable 5. Incidence Rate (IR, per 10 000 Person-Years) and Hazard Ratio (HRs) With 95% CIs of Hospital-Treated Infectious Diseases or Other Psychiatric Disorders Associated With Surgical Removal of Tonsils or Adenoids in the Population-Matched and Sibling-Matched Cohorts [file jamanetwopen-e2449807-s001.pdf]

Supplemental Online Content

Xiao X, Yang F, Yin L, et al. Stress-related disorders among young individuals with surgical removal of tonsils or adenoids. *JAMA Netw Open*. 2024;7(12):e2449807.
doi:10.1001/jamanetworkopen.2024.49807

eTable 1. Swedish Revisions of the *International Classification of Diseases* (*ICD*) Codes Used to Identify the Indications for Surgical Removal of Tonsils or Adenoids

eTable 2. Incidence Rate (IR, per 10 000 Person-Years) and Hazard Ratio (HRs) With 95% CIs of Stress-Related Disorders Associated With Surgical Removal of Tonsils or Adenoids, Sensitivity Analyses With Different Lag Periods

eTable 3. Incidence Rate (IR, per 10 000 Person-Years) and Hazard Ratio (HRs) With 95% CIs of Stress-Related Disorders Associated With Surgical Removal of Tonsils or Adenoids in the Sibling-Matched Cohort, Stratified Analysis

eTable 4. Incidence Rate (IR, per 10 000 Person-Years) and Hazard Ratio (HRs) With 95% CIs of Stress-Related Disorders Associated With Surgical Removal of Tonsils or Adenoids in the Population-Matched and Sibling-Matched Cohorts, Analysis by Potential Indication for Surgery

eTable 5. Incidence Rate (IR, per 10 000 Person-Years) and Hazard Ratio (HRs) With 95% CIs of Hospital-Treated Infectious Diseases or Other Psychiatric Disorders Associated With Surgical Removal of Tonsils or Adenoids in the Population-Matched and Sibling-Matched Cohorts

This supplemental material has been provided by the authors to give readers additional information about their work.

© 2024 Xiao X et al. *JAMA Network Open*.

eTable 1. Swedish Revisions of the *International Classification of Diseases* (*ICD*) Codes Used to Identify the Indications for Surgical Removal of Tonsils or Adenoids

**Indications**	**ICD-8**	**ICD-9**	**ICD-10**
Hypertrophy of tonsils and adenoids	500	474B	J35.1, J35.2, J35.3
Other diseases of tonsils and adenoids		474C, 474W, 474X	J35.8, J35.9
Chronic tonsilitis, pharyngitis and nasopharyngitis, or peritonsillar abscess	501, 502	472B, 473C, 474A, 475X	J31.1, J31.2, J35.0, J36
Sleep disorders or dyspnea and respiratory abnormalities	306.4, 783.2, 783.6	780F, 786A, 786B	G47, R06.0, R06.1, R06.3, R06.5

© 2024 Xiao X et al. *JAMA Network Open*.

eTable 2. Incidence Rate (IR, per 10 000 Person-Years) and Hazard Ratio (HRs) With 95% CIs of Stress-Related Disorders Associated With Surgical Removal of Tonsils or Adenoids, Sensitivity Analyses With Different Lag Periods

Lag period (years)	Individuals without surgery		Individuals with surgery	
	N of cases/IR	HR (95% CI)*	N of cases/IR	HR (95% CI)*
**Population-matched cohort**				
1	20,670/23.4	Ref	2958/33.7	1.40 (1.35-1.46)
2	19,451/24.2	Ref	2747/34.3	1.38 (1.33-1.44)
3	18,206/24.9	Ref	2533/34.9	1.37 (1.31-1.42)
**Sibling-matched cohort**				
1	1699/23.3	Ref	1600/31.3	1.32 (1.23-1.43)
2	1595/24.1	Ref	1483/32.0	1.31 (1.21-1.42)
3	1501/25.2	Ref	1359/32.5	1.27 (1.17-1.38)

*Analysis of the population-matched cohort used time since follow-up as the underlying time scale and was conditioned on sex, birth year, and calendar date at the start of follow-up and additionally adjusted for parental educational attainment and parental history of stress-related disorders at the start of follow-up; analysis of the sibling-matched cohort used time since follow-up as the underlying time scale and was conditioned on family identifiers and calendar date at the start of follow-up and additionally adjusted for sex and age at the start of follow-up. Parental educational attainment and history of stress-related disorders at the start of follow-up were not adjusted for in the sibling-matched cohort as the exposed person and their unexposed full siblings shared this information at the start of follow-up.

© 2024 Xiao X et al. *JAMA Network Open*.

eTable 3. Incidence Rate (IR, per 10 000 Person-Years) and Hazard Ratio (HRs) With 95% CIs of Stress-Related Disorders Associated With Surgical Removal of Tonsils or Adenoids in the Sibling-Matched Cohort, Stratified Analysis

	Individuals without surgery		Individuals with surgery	
**Characteristics**	**N of cases/IR**	**HR (95% CI)***	**N of cases/IR**	**HR (95% CI)***
**Sex**				
Male	644/15.1	Ref	470/17.4	1.28 (1.08-1.52)
Female	1166/30.8	Ref	1272/43.7	1.37 (1.22-1.53)
**Age at the start of follow-up (years)**				
1-6	327/12.9	Ref	251/15.2	1.01 (0.76-1.31)
7-12	497/20.6	Ref	375/22.2	1.26 (1.02-1.56)
13-18	508/29.7	Ref	678/50.1	1.32 (1.10-1.59)
19-25	403/34.0	Ref	382/45.4	1.30 (1.05-1.61)
26-	75/38.3	Ref	56/75.6	2.13 (1.12-4.05)
**Time since the start of follow-up (years)**				
0-10	996/12.4	Ref	1073/19.1	1.56 (1.42-1.72)
11-20	692/11.4	Ref	543/12.7	1.07 (0.94-1.21)
21-	121/4.7	Ref	125/6.4	1.41 (1.06-1.88)
**Parental educational attainment**				
≤9 years	117/33.1	Ref	119/52.1	1.38 (1.08-1.75)
>9-12 years	1082/26.3	Ref	994/34.9	1.33 (1.21-1.46)
>12 years	610/17.1	Ref	627/24.7	1.38 (1.21-1.57)
**Parental history of stress-related disorders**				
No	1403/19.4	Ref	1381/29.1	1.32 (1.23-1.43)
Yes	407/50.7	Ref	361/68.9	1.60 (1.22-2.11)

*Analysis of the sibling-matched cohort used time since follow-up as the underlying time scale and was conditioned on family identifiers and calendar date at the start

© 2024 Xiao X et al. *JAMA Network Open*.

eTable 4. Incidence Rate (IR, per 10 000 Person-Years) and Hazard Ratio (HRs) With 95% CIs of Stress-Related Disorders Associated With Surgical Removal of Tonsils or Adenoids in the Population-Matched and Sibling-Matched Cohorts, Analysis by Potential Indication for Surgery

	**Individuals without surgery**		**Individuals with surgery**	
	**N of cases/IR**	**HR (95% CI)***	**N of cases/IR**	**HR (95% CI)***
**Population-matched cohort**				
Chronic tonsilitis, pharyngitis and nasopharyngitis, or peritonsillar abscess	12,573/26.8	Ref	1855/39.8	1.45 (1.38-1.53)
Hypertrophy of tonsils or adenoids	8093/18.6	Ref	1163/26.8	1.39 (1.31-1.48)
Sleep disorders or dyspnea and respiratory abnormalities	97/16.8	Ref	29/50.5	2.91 (1.92-4.42)
Other diseases of tonsils or adenoids	598/20.5	Ref	91/31.4	1.54 (1.23-1,91)
No clear indication	501/20.2	Ref	56/22.6	1.10 (0.84-1.45)
**Sibling-matched cohort**				
Chronic tonsilitis, pharyngitis and nasopharyngitis, or peritonsillar abscess	970/23.9	Ref	1010/36.4	1.43 (1.29-1.57)
Hypertrophy of tonsils or adenoids	739/21.2	Ref	653/26.2	1.28 (1.14-1.44)
Sleep disorders or dyspnea and respiratory abnormalities	15/29.1	Ref	16/45.9	1.73 (0.83-3.64)
Other diseases of tonsils or adenoids	49/20.5	Ref	45/26.8	1.52 (0.94-2.45)
No clear indication	37/19.1	Ref	18/12.8	0.64 (0.34-1.23)

*Analysis of the population-matched cohort used time since follow-up as the underlying time scale and was conditioned on sex, birth year, and calendar date at the start of follow-up and additionally adjusted for parental educational attainment and parental history of stress-related disorders at the start of follow-up; analysis of the sibling-matched cohort used time since follow-up as the underlying time scale and was conditioned on family identifiers and calendar date at the start of follow-up and additionally adjusted for sex and age at the start of follow-up. Parental educational attainment and history of stress-related disorders at the start of follow-up were not adjusted for in the sibling-matched cohort as the exposed person and their unexposed full siblings shared this information at the start of follow-up.

© 2024 Xiao X et al. *JAMA Network Open*.

eTable 5. Incidence Rate (IR, per 10 000 Person-Years) and Hazard Ratio (HRs) With 95% CIs of Hospital-Treated Infectious Diseases or Other Psychiatric Disorders Associated With Surgical Removal of Tonsils or Adenoids in the Population-Matched and Sibling-Matched Cohorts

	**Individuals without surgery**		**Individuals with surgery**	
	**N of cases/IR**	**HR (95% CI)***	**N of cases/IR**	**HR (95% CI)***
**Population-matched cohort**				
Infectious disease	228,246/283.5	Ref	32,688/459.0	1.61 (1.59-1.63)
Other psychiatric disorders	122,987/137.3	Ref	15,916/183.0	1.31 (1.29-1.33)
**Sibling-matched cohort**				
Infectious disease	21,943/340.3	Ref	19,012/451.3	1.31 (1.28-1.34)
Other psychiatric disorders	11,460/154.4	Ref	9197/180.3	1.24 (1.20-1.27)

*Analysis of the population-matched cohort used time since follow-up as the underlying time scale and was conditioned on sex, birth year, and calendar date at the start of follow-up and additionally adjusted for parental educational attainment and parental history of stress-related disorders at the start of follow-up; analysis of the sibling-matched cohort used time since follow-up as the underlying time scale and was conditioned on family identifiers and calendar date at the start of follow-up and additionally adjusted for sex and age at the start of follow-up. Parental educational attainment and history of stress-related disorders at the start of follow-up were not adjusted for in the sibling-matched cohort as the exposed person and their unexposed full siblings shared this information at the start of follow-up.

© 2024 Xiao X et al. *JAMA Network Open*.
