# Supplementary material for: Stress-Related Disorders Among Young Individuals With Surgical Removal of Tonsils or Adenoids
Source: JAMA Netw Open. 2024 Dec 9;7(12):e2449807. doi: 10.1001/jamanetworkopen.2024.49807 (PMC11629130; doi:10.1001/jamanetworkopen.2024.49807)
Supplement: Supplement 2. — Data Sharing Statement [file jamanetwopen-e2449807-s002.pdf]

Data Sharing Statement

Xiao. Stress-Related Disorders Among Young Individuals With Surgical Removal of Tonsils or Adenoids. *JAMA Netw Open*. Published December 09, 2024.
doi:10.1001/jamanetworkopen.2024.49807

Data
Data available: No

Additional Information
Explanation for why data not available: Data used in the present study are not directly sharable due to European and Swedish laws.
